# Does the mini-open technique still have a role in the modern management of pediatric tibial eminence fractures?

**DOI:** 10.3389/fsurg.2026.1825209

**Published:** 2026-08-31

**Authors:** Michela Florio, Marco Giordano, Martina Marsiolo, Luca Basiglini, Angelo Gabriele Aulisa, Silvia Careri, Francesco Falciglia

**Affiliations:** 1 Bambino Gesù Children’s Hospital (IRCCS), Rome, Italy; 2 Department of Human, Social and Health Sciences, University of Cassino and Southern Lazio, Cassino, Italy

**Keywords:** mini-open technique, pediatric knee, physeal-sparing fixation, tibial eminence fracture, transosseous suture fixation

## Abstract

**Background:**

Tibial eminence fractures are uncommon pediatric injuries that often require surgical fixation. Although arthroscopic fixation is widely adopted, the role of mini-open physeal-sparing techniques remains incompletely defined. This study evaluated the medium- and long-term clinical and radiographic outcomes of pediatric patients treated with this technique for displaced tibial eminence fractures.

**Methods:**

A retrospective study was conducted on 26 consecutive patients (mean age 12.1 ± 2.3 years) treated between 2017 and 2019 with a mini-open physeal-sparing transosseous suture technique. Clinical and radiographic follow-up averaged 7.6 ± 2.9 years. Outcomes included fracture union, range of motion, residual anterior laxity, return to sport, complications, and patient-reported outcome measures (Lysholm Knee Score, subjective International Knee Documentation Committee score, and Tegner Activity Scale). Non-parametric statistical analyses were performed to evaluate correlations between baseline characteristics and clinical outcomes, as well as differences between patients with and without residual anterior laxity.

**Results:**

Fracture union was achieved in all patients at a mean of 35.8 ± 6.4 days. No cases of infection, nonunion, arthrofibrosis, growth disturbance, limb-length discrepancy, or fixation failure were observed. At final follow-up, no patient had persistent range-of-motion limitation, and all returned to their pre-injury level of sports activity after a mean of 5.1 ± 1.1 months. The median Lysholm score was 99 (IQR 98–100), the median subjective IKDC score was 97.8 (IQR 96.5–99.4), and the median Tegner Activity Score was 8.5 (IQR 8–9). Residual anterior laxity was detected in 11 patients (42.3%), although only one reported subjective instability. Patients with residual anterior laxity had lower Lysholm scores. Although final Tegner Activity Scores were also lower, this reflected lower pre-injury activity levels, as all patients returned to their individual pre-injury Tegner Activity Score. Subjective IKDC scores were comparable between groups.

**Conclusions:**

This technique provided reliable fracture healing, excellent functional outcomes, complete return to pre-injury activity levels, and a low complication rate at medium- to long-term follow-up. Although arthroscopic fixation is currently the most commonly used technique, the mini-open approach represents a safe and effective alternative for selected displaced pediatric tibial eminence fractures, particularly when fracture characteristics or local resources limit the use of arthroscopy.

## Introduction

Tibial intercondylar eminence fractures (TEFs) are uncommon injuries in children and adolescents, with an estimated incidence of approximately 3 per 100,000 pediatric fractures (1–3). These injuries involve the tibial insertion of the anterior cruciate ligament (ACL) and are considered the pediatric counterpart of ACL injuries in adults. They typically occur during sports activities or high-energy trauma through a combination of hyperextension and rotational forces acting on the knee (4, 5). If inadequately treated, displaced fractures may result in malunion, loss of knee extension, residual instability, or persistent functional impairment (6–9).

Treatment is primarily guided by fracture displacement. Non-operative management is generally accepted for minimally displaced fractures, whereas displaced fractures usually require surgical reduction and fixation to restore joint congruity and knee stability (6, 7, 10). Historically, surgical treatment often involved extensive dissection and manipulation of the physis, increasing the risk of growth disturbances and joint deformities. With the development of physeal-sparing techniques and improved understanding of pediatric knee biomechanics, less invasive approaches have been increasingly adopted. Arthroscopic reduction and internal fixation have become the most widely used techniques because they allow direct visualization of the joint and treatment of associated intra-articular lesions (11–13).

However, arthroscopic procedures may not always be feasible. Open and mini-open techniques remain valid alternatives, particularly in cases of complex fracture patterns, soft-tissue interposition, or when arthroscopic expertise and resources are limited (14–20).

Despite the widespread adoption of arthroscopic techniques, the role of mini-open physeal-sparing fixation remains incompletely defined. Long-term data on fracture healing, functional recovery, return to sport, residual laxity, and growth-related complications are still limited (21, 22). Therefore, the aim of this study was to evaluate the medium- and long-term clinical and radiographic outcomes of pediatric patients treated with a mini-open physeal-sparing technique for tibial eminence fractures, with particular attention to fracture union, knee stability, functional outcomes, return to sport, complications, and growth disturbances.

## Methods

This retrospective study was conducted between January 2017 and December 2019 after approval by the Institutional Review Board. Written informed consent was obtained from all patients and their legal guardians. A total of 32 patients with tibial intercondylar eminence fractures were initially identified and assessed for eligibility. Six patients were excluded because they presented with non-displaced (minimally displaced) or stable fracture patterns managed conservatively. Therefore, 26 patients who met the inclusion criteria and underwent surgical treatment with the mini-open physeal-sparing technique were included in the final analysis. Fractures were classified preoperatively using the Zaricznyj-modified Meyers and McKeever classification system. The inclusion criteria were patients aged between 8 and 16 years with type II posterior-hinged fractures that exhibited displacement, as well as type III or IV isolated tibial spine fractures.

Patients with fused growth plates at the time of trauma, those with additional intra-knee pathologies or fractures, pre-existing cartilage or meniscal damage, as well as prior ligament ruptures, and those who lacked regular follow-up were excluded from the study. All patients underwent standard anteroposterior and lateral knee radiography both preoperatively and postoperatively, along with a diagnostic preoperative CT scan to improve the accuracy of assessing fracture displacement and classification and to facilitate preoperative planning. Preoperative computed tomography (CT) was routinely performed because it could be obtained rapidly in the emergency setting, allowing accurate assessment of fracture morphology while minimizing the time from admission to surgical treatment. Although magnetic resonance imaging (MRI) represents the imaging modality of choice for comprehensive preoperative assessment, particularly for the evaluation of associated soft-tissue injuries, it was not routinely performed in our institution because it could not be obtained rapidly in the emergency setting and was associated with substantially longer waiting times, potentially delaying surgical treatment. All patients underwent surgical treatment with the mini-open physeal-sparing technique. A clinical evaluation was conducted to determine the range of motion (ROM) and residual instability, using an objective rating scale (IKDC checklist). Patients were also evaluated using subjective rating scales to assess functional outcomes and overall satisfaction (International Knee Documentation Committee Questionnaire, Lysholm Knee Scoring Scale, and Tegner Activity Scale). We also assessed for complications, including nonunion, residual laxity, limb discrepancy, and arthrofibrosis. Clinical and radiographic assessments were performed at 7 days, 30 days, 3 months, and 6 months post-surgery. The cast was removed 30 days after the procedure, and replaced with a brace. Following cast removal, all patients began physiotherapy, which included isometric exercises and gradual joint recovery program. At six weeks, weight-bearing activities and swimming were permitted, along with exercises on a high-saddle exercise bike that limited knee flexion to less than 90°. Patients were allowed to return to sports activities 5–6 months after injury.

### Outcome assessment

Radiographic union was defined as the presence of bridging trabecular bone across the fracture site and absence of fracture displacement on standard radiographs.

Residual anterior laxity was assessed clinically using the Lachman test. The Lachman test was performed at the final follow-up by an experienced pediatric orthopedic surgeon and was compared with the contralateral knee in all patients. Residual laxity was graded according to the objective IKDC classification.

Arthrofibrosis was defined as a persistent limitation of knee range of motion exceeding 10° of extension loss or 20° of flexion loss at final follow-up.

Limb-length discrepancy was defined as a side-to-side difference greater than 1 cm and was assessed clinically using block testing and tape measurement during follow-up examinations. Standing long-leg radiographs and magnetic resonance imaging were obtained only when clinically indicated.

Growth disturbance was defined as any clinically or radiographically evident angular deformity or limb-length discrepancy attributable to physeal injury. At the final follow-up, all patients underwent standardized clinical evaluation, supplemented by radiographic assessment when clinically indicated. Skeletal maturity was documented when reached, whereas patients who had not yet reached skeletal maturity underwent the same evaluation for evidence of growth disturbance.

Other complications included infection, fixation failure and secondary displacement.

### Statistical analysis

Statistical analyses were performed using IBM SPSS Statistics version 26.0 (IBM Corp., Armonk, NY, USA). The normality of continuous variables was assessed using the Shapiro–Wilk test. Age at injury (W = 0.950, *p* = 0.236) and initial fracture displacement (W = 0.932, *p* = 0.085) were normally distributed, whereas follow-up duration (W = 0.906, *p* = 0.021), Lysholm score (W = 0.700, *p* < 0.001), subjective IKDC score (W = 0.748, *p* < 0.001), Tegner Activity Score (W = 0.712, *p* < 0.001), and fracture healing time (W = 0.749, *p* < 0.001) showed non-normal distributions (Table 1). Therefore, non-parametric statistical methods were used for comparative analyses. Continuous variables are presented as mean ± standard deviation (SD) for descriptive purposes, whereas the results of the statistical analyses are reported as median with interquartile range (IQR). All reported values were rounded to one decimal place, where applicable. Given the exploratory nature of these *post hoc* comparisons, confidence intervals were not calculated; instead, rank-biserial effect sizes are reported to describe the magnitude of the observed between-group differences. The results of the normality assessment are summarized in Table 1.

**Table 1:** Assessment of normality of continuous variables using the Shapiro–Wilk test.

Variable	Shapiro–Wilk W	*p* value	Distribution
Age at injury (years)	0.950	0.236	Normal
Initial fracture displacement (mm)	0.932	0.085	Normal
Follow-up duration (years)	0.906	0.021	Non-normal
Lysholm score	0.700	<0.001	Non-normal
Subjective IKDC score	0.748	<0.001	Non-normal
Tegner Activity Score	0.712	<0.001	Non-normal
Fracture healing time (days)	0.749	<0.001	Non-normal

Normality testing was performed to determine the appropriate statistical approach. Variables that did not meet the assumption of normality were analyzed using non-parametric statistical methods.

Pre-injury and final Tegner Activity Scores were identical in all patients, indicating complete recovery of the pre-injury activity level. As no paired differences were observed, statistical comparison using the Wilcoxon signed-rank test was not applicable.

Differences in clinical outcomes between patients with and without residual anterior laxity (positive versus negative Lachman test) were evaluated using the Mann–Whitney U test. Associations between age at injury, initial fracture displacement, follow-up duration, and clinical outcome measures (Lysholm score, subjective IKDC score, Tegner Activity Score, and fracture healing time) were assessed using Spearman's rank correlation coefficient. Categorical variables were compared using Fisher's exact test when appropriate. Effect sizes for Mann–Whitney U tests were calculated using the rank-biserial correlation.

Given the retrospective design and relatively small sample size, all statistical analyses were considered exploratory and should be interpreted accordingly. Statistical significance was defined as a two-sided *p* value < 0.05.

### Surgical technique

All procedures were performed under general anesthesia with the patient in the supine position and a pneumatic tourniquet applied to the proximal thigh. Following standard skin preparation and sterile draping, a mini-open anteromedial approach was used.

A skin incision of approximately 4–5 cm was made, extending from the distal parapatellar region to the proximal tibia. Compared with the traditional open approach, this limited incision, without patellar eversion, allowed direct visualization of the fracture site while minimizing soft-tissue dissection and surgical morbidity. The joint capsule was carefully incised, and the fracture fragment was identified. Any interposed soft tissue, including the meniscus or the transverse intermeniscal ligament, when present, was removed to facilitate anatomical reduction.

The avulsed tibial eminence fragment was anatomically reduced under direct visualization and, when necessary, temporarily stabilized with a Kirschner wire. Definitive fixation was performed using a physeal-sparing transosseous suture technique. Non-absorbable high-strength sutures were passed through the substance of the anterior cruciate ligament near its tibial insertion and subsequently shuttled, using an Endo Close device, through tibial bone tunnels created distal to the proximal tibial physis, thereby preserving physeal integrity.

**Figure 1:**
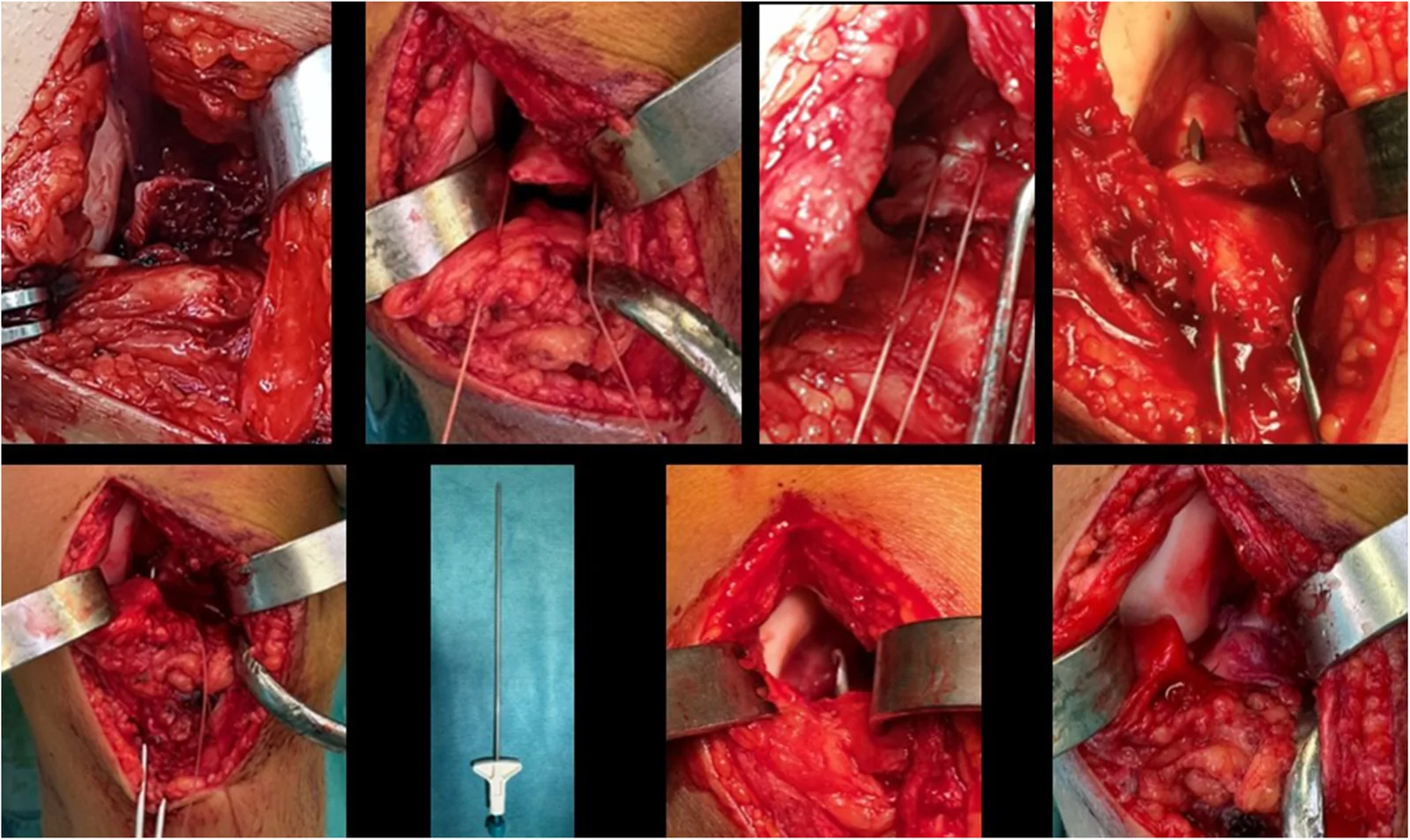
Mini-open physeal-sparing surgical technique. Sequential intraoperative photographs illustrating the main steps of the mini-open physeal-sparing fixation technique for pediatric tibial intercondylar eminence fractures.

Following confirmation of anatomical reduction and satisfactory fixation, the sutures were tied over a cortical bone bridge on the anterior tibial cortex. Stability of the fixation and restoration of ACL tension were assessed intraoperatively through knee range-of-motion testing and Lachman examination. Final fluoroscopic images were obtained to confirm fracture reduction and hardware-free fixation (Figure 1).

The wound was irrigated and closed in layers. Postoperatively, the knee was immobilized according to the rehabilitation protocol, with progressive recovery of range of motion and weight-bearing introduced during follow-up.

## Results

**Figure 2:**
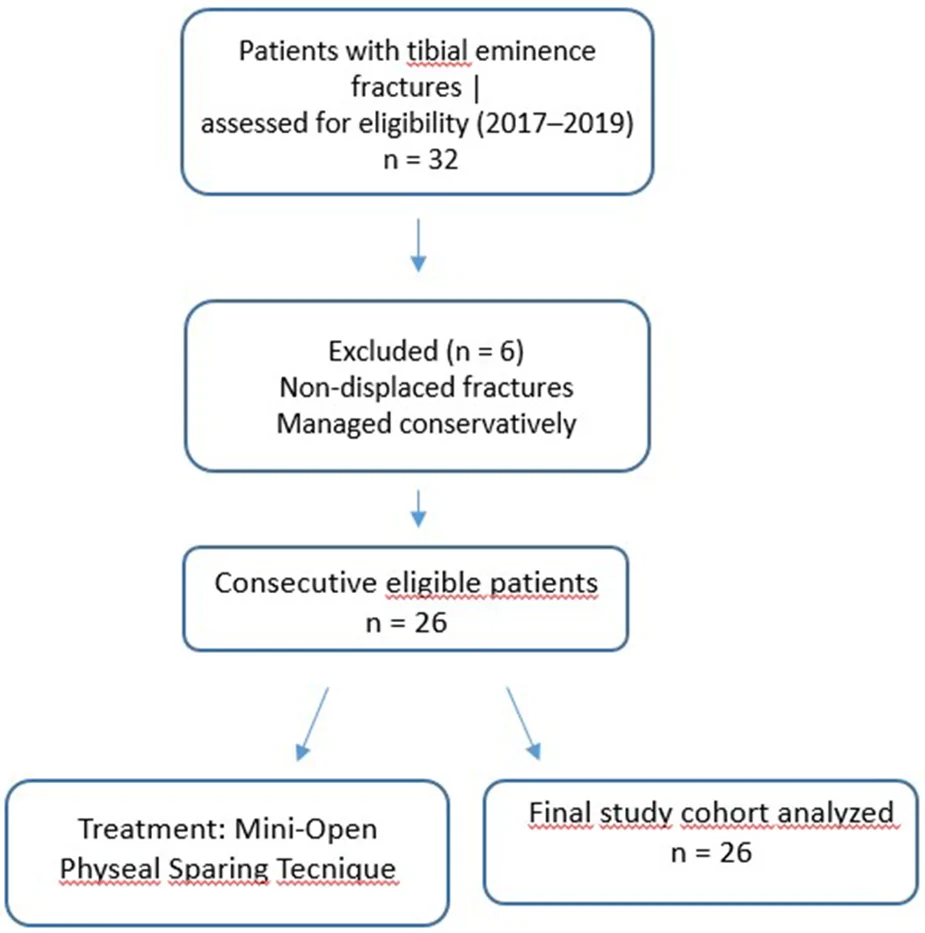
Flow diagram of patient enrollment. Thirty-two patients were assessed for eligibility; six were excluded due to non-displaced or stable fractures treated non-operatively. Twenty-six patients were included in the final analysis.

During the study period, 32 consecutive patients with tibial intercondylar eminence fractures were assessed for eligibility. Six patients were excluded because they had non-displaced or stable fractures that were successfully managed non-operatively. The remaining 26 patients met the eligibility criteria, underwent mini-open physeal-sparing fixation, and were included in the final analysis (Figure 2).

**Figure 3:**
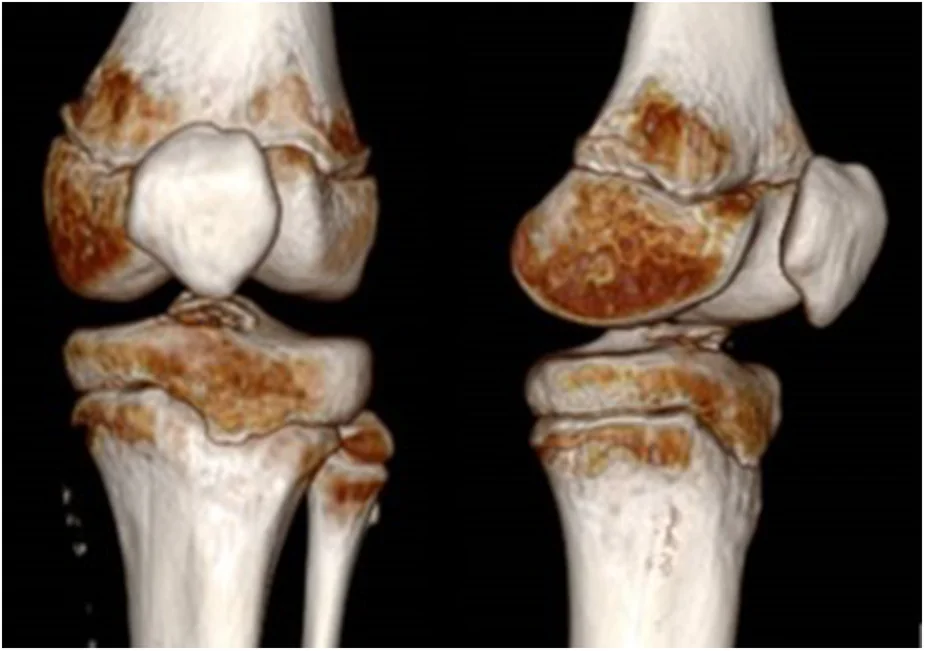
Three-dimensional computed tomography (3D CT). Preoperative three-dimensional CT reconstructions in coronal and sagittal views demonstrating a displaced Meyers–McKeever type II tibial intercondylar eminence fracture.

**Figure 4:**
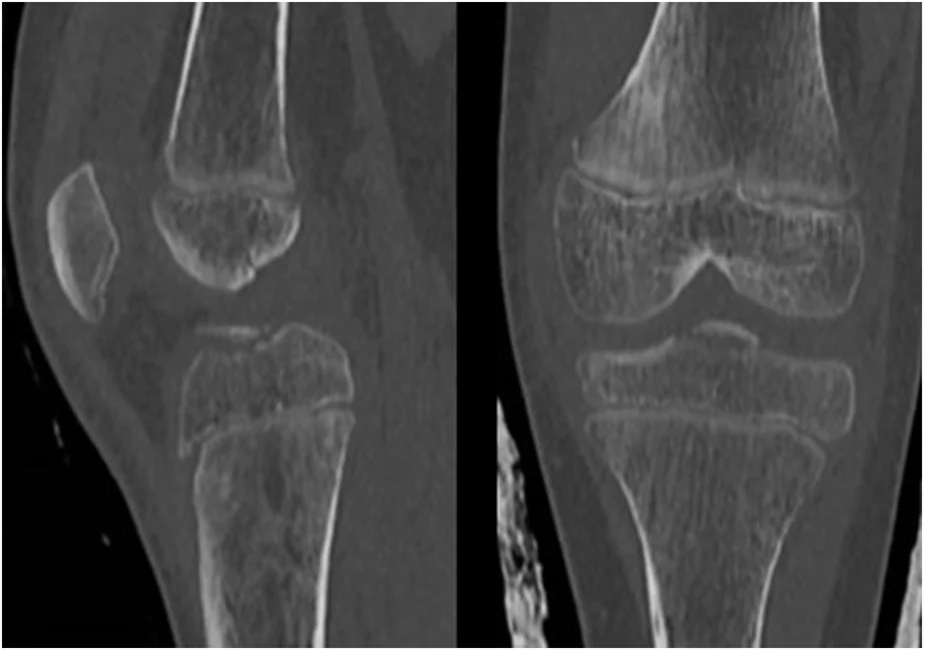
Computed tomography (CT). Preoperative coronal and sagittal CT images of the same patient shown in Figure 3, confirming the displaced Meyers–McKeever type II fracture.

All patients were aged 16 years or younger, with a mean age of 12.1 ± 2.3 years at the time of injury. The study cohort consisted of 14 females and 12 males. The right knee was involved in 20 patients and the left knee in 6. Sports-related injuries occurred in 13 patients (50%), including skiing (*n* = 7) and cycling (*n* = 6). Domestic falls accounted for 7 injuries (27%), whereas vehicle-related accidents were reported in 6 patients (23%). According to the Zaricznyj-modified Meyers and McKeever classification, two fractures were classified as type II (Figures 3, 4), thirteen as type III, and eleven as type IV**.** Baseline demographic and injury characteristics are summarized in Table 2. Detailed patient-level demographic, injury, and clinical outcome data are presented in Table 3.

**Table 2:** Baseline demographic and injury characteristics of the study cohort.

Characteristic	Category	*n*
Sex	Male	12
	Female	14
Side	Right	20
	Left	6
Circumstance	Sports-related injury	13
	Domestic fall	7
	Vehicle-related accident	6
Type of fracture	Type II	2
	Type III	13
	Type IV	11

Distribution of patients according to sex, affected side, mechanism of injury, and fracture type according to the Zaricznyj-modified Meyers and McKeever classification.

**Table 3:** Individual patient demographic, injury, and outcome data**.**

Patient#	Age at Injury (y)	Initial displacement (mm)	Years of follow-up	Arthrofibrosis or other complications	ROM deficiency at final follow-up	Residual laxity (Lachman test)	Return to Previous level of activity	Lysholm score	IKDC (objective)	IKDC (subjective)	TAS	Fracture healing time
1	9	2.1	4	no	no	no	yes	100	A	98.8	9/9	30
2	10	2.8	5	no	no	no	yes	100	A	100	9/9	40
3	8	4.3	4	no	no	no	yes	100	A	97.3	8/8	30
4	11	8.4	8	no	no	Yes (+)	yes	99	B	98	6/6	40
5	12	11.5	7	no	no	no	yes	98	A	100	8/8	30
6	15	13.2	9	no	no	Yes (++)	yes	96	C	87.6	6/6	30
7	14	10.4	12	no	no	Yes (+)	yes	88	B	96	8/8	40
8	12	11.8	5	no	no	Yes (+)	yes	99	B	97.4	8/8	30
9	11	3.4	6	no	no	no	yes	96	A	96.5	9/9	30
10	9	5.8	8	no	no	no	yes	100	A	98	9/9	30
11	10	8.9	5	no	no	Yes (+)	yes	100	B	96.7	6/6	40
12	11	12.5	6	no	no	Yes (+)	yes	100	B	97.3	6/6	40
13	14	11.6	11	no	no	Yes (+)	yes	97	B	99.5	8/8	40
14	13	7.5	10	no	no	no	yes	100	A	98.8	9/9	30
15	11	5.3	7	no	no	no	yes	99	A	99.4	9/9	30
16	15	14.4	4	no	no	Yes (+)	yes	93	B	100	9/9	40
17	16	16.5	5	no	no	Yes (+)	yes	95	B	88.5	8/8	40
18	16	13.4	7	no	no	no	yes	100	A	97.6	9/9	50
19	14	11.6	12	no	no	no	yes	100	A	89	9/9	40
20	15	12.3	13	no	no	Yes (+)	yes	98	B	94.5	8/8	40
21	9	3.5	11	no	no	no	yes	100	A	96.8	9/9	50
22	11	2.7	13	no	no	no	yes	100	A	100	9/9	30
23	10	3.2	8	no	no	no	yes	99	A	99.4	8/8	30
24	12	8.5	7	no	no	Yes (+)	yes	99	B	98.3	8/8	30
25	13	6.8	6	no	no	no	yes	98	A	87.4	9/9	30
26	14	2	5	no	no	no	yes	98	A	98.4	9/9	40

Patient-level data including age at injury, fracture characteristics, follow-up duration, clinical findings, functional outcomes, return to sport, and fracture healing time. Tegner activity score is reported as pre-injury/final follow-up.

**Figure 5:**
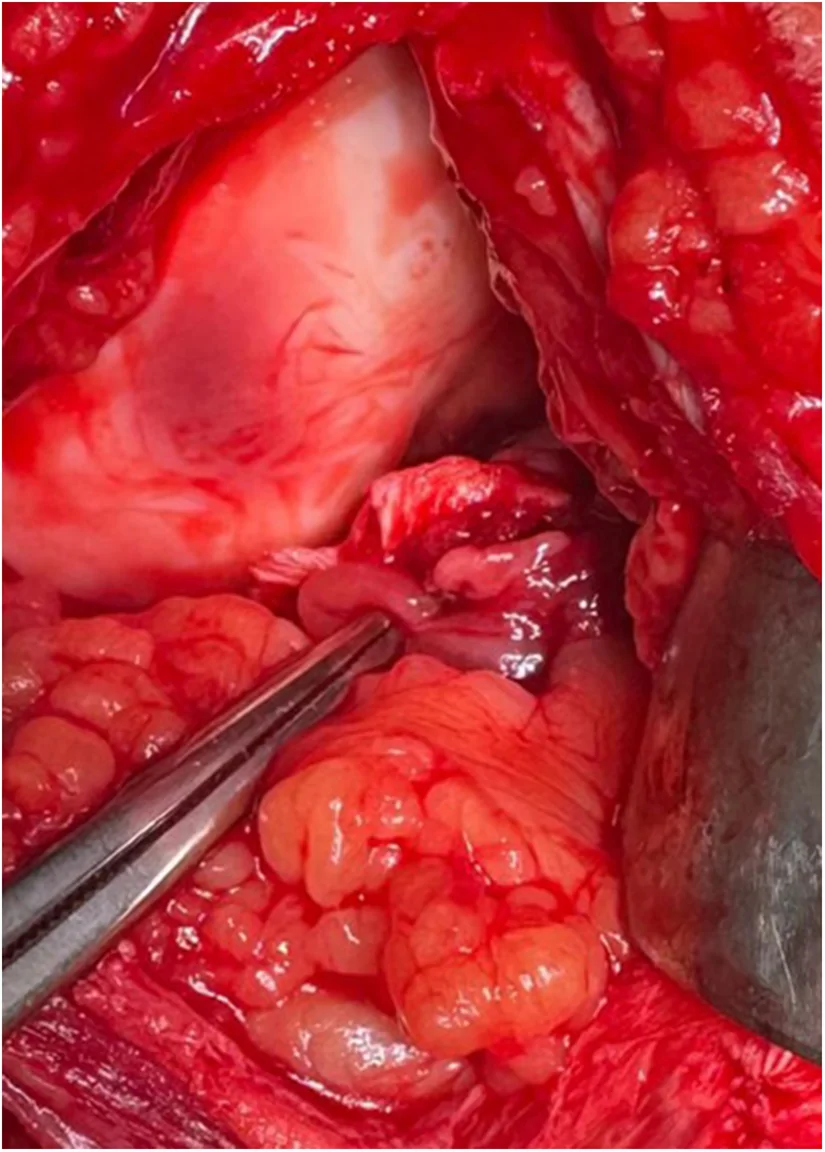
Intraoperative finding. Intraoperative photograph obtained through the mini-open arthrotomy demonstrating interposition of the anterior horn of the medial meniscus within the fracture site, preventing anatomical reduction.

**Figure 6:**
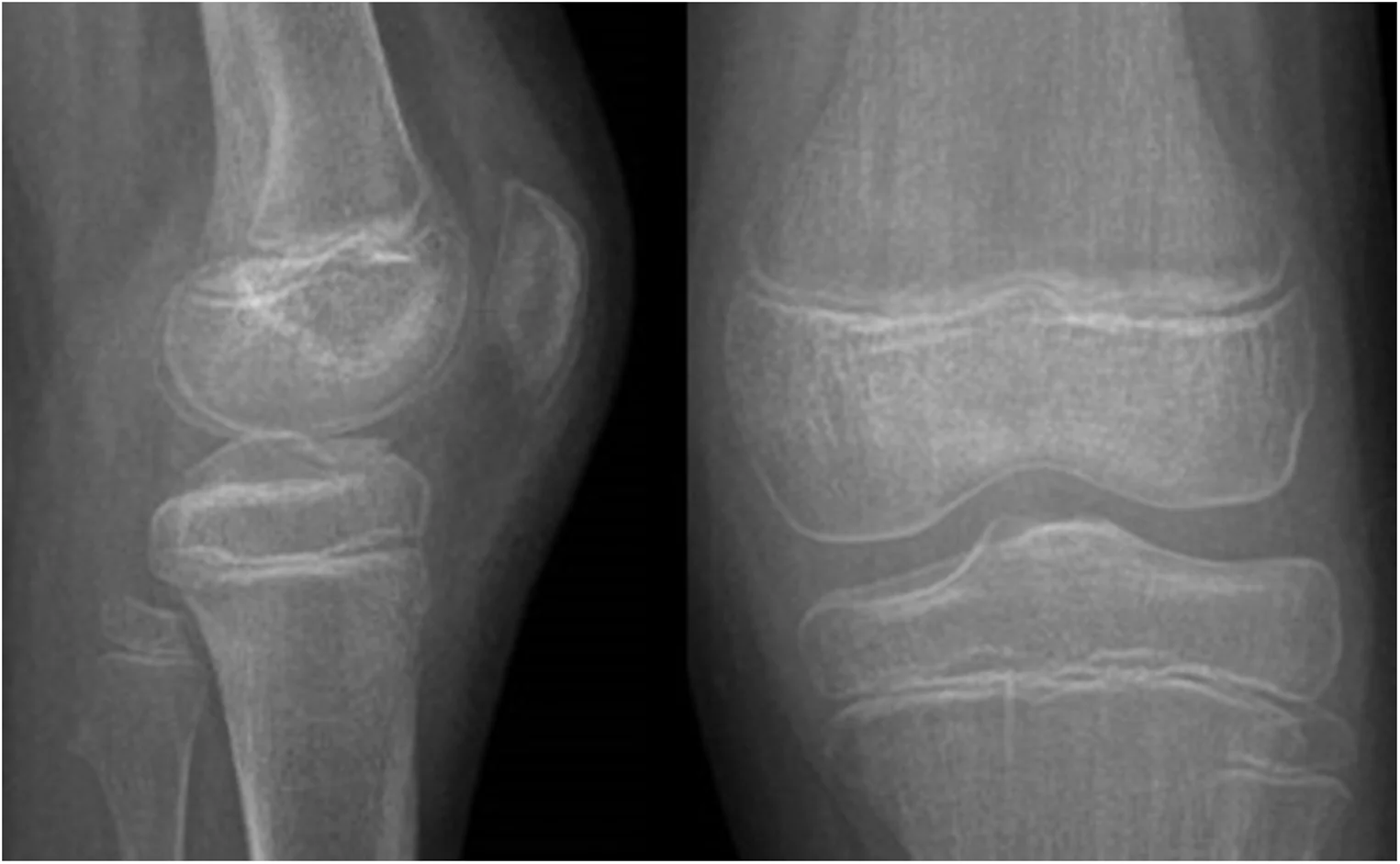
Postoperative radiographic follow-up. Anteroposterior and lateral radiographs obtained at final follow-up demonstrating complete fracture union and anatomical reduction of the tibial eminence.

**Figure 7:**
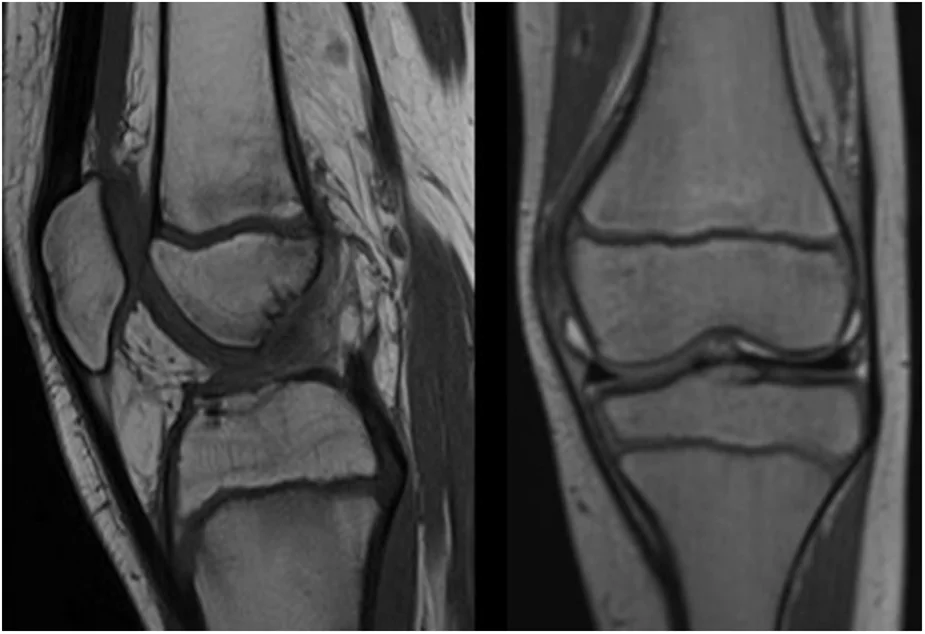
Postoperative magnetic resonance imaging (MRI). MRI images obtained during follow-up demonstrating complete fracture healing, restoration of ACL insertion, and absence of residual fragment displacement or intra-articular soft-tissue interposition.

The mean clinical follow-up was 7.6 ± 2.9 years. No patient demonstrated residual range-of-motion limitation at final follow-up, and no cases of infection, nonunion, growth disturbance, limb-length discrepancy, arthrofibrosis, or fixation failure were observed. At final follow-up, 22 of the 26 patients had reached skeletal maturity, whereas 4 patients remained skeletally immature. None of the patients showed clinical or radiographic evidence of growth disturbance at the final evaluation. One patient presented with interposition of the anterior horn of the medial meniscus within the fracture site, which was identified and treated during surgery (Figure 5). The mean fracture healing time was 35.8 ± 6.4 days (Figures 6, 7). Six patients presented with transient flexion deficits during the early postoperative period (7 days, 30 days, and 3 months), measuring 10°, 5°, and 15°, respectively. All deficits resolved completely by the final follow-up, and no extension deficits were observed.

The mean time to return to sport was 5.1 ± 1.1 months. The mean subjective IKDC score was 96.6 ± 3.9, the mean Lysholm Knee Score was 98.2 ± 2.8, and the mean Tegner Activity Score was 8.2 ± 1.1. Residual anterior laxity, defined as a positive Lachman test, was observed in 11 of 26 patients (42.3%). Of these, ten patients demonstrated mild residual laxity (IKDC grade B), whereas one patient demonstrated marked laxity (IKDC grade C). The remaining fifteen patients had a negative Lachman test and were classified as IKDC grade A. Only one patient reported subjective instability during sports activities, although no limitations in daily activities were reported**.**

### Statistical analysis results

The median Lysholm score at final follow-up was 99 (IQR 98–100), the median subjective IKDC score was 97.8 (IQR 96.5–99.4), the median Tegner Activity Score was 8.5 (IQR 8–9), and the median fracture healing time was 35 days (IQR 30–40 days).

Pre-injury and final Tegner Activity Scores were identical in all patients, indicating complete recovery of the pre-injury activity level. As no paired differences were observed, statistical comparison using the Wilcoxon signed-rank test was not applicable.

Spearman correlation analysis demonstrated a significant negative correlation between age at injury and Lysholm score at final follow-up (*ρ* = −0.60, *p* = 0.001), indicating slightly lower functional scores in older patients. Initial fracture displacement was negatively correlated with the final Tegner Activity Score (*ρ* = −0.45, *p* = 0.022), whereas no significant correlations were found between fracture displacement and Lysholm score (*p* = 0.073), subjective IKDC score (*p* = 0.081), or fracture healing time (*p* = 0.084). Likewise, age at injury was not significantly associated with subjective IKDC score (*p* = 0.208), Tegner Activity Score (*p* = 0.776), or fracture healing time (*p* = 0.103). No significant correlations were observed between follow-up duration and any clinical outcome measure (Table 4).

**Table 4:** Spearman correlation analysis between baseline variables and clinical outcomes.

Baseline variable	Outcome	Spearman *ρ*	*p* value
Age at injury	Lysholm score	−0.60	0.001
Age at injury	Subjective IKDC score	−0.26	0.208
Age at injury	Tegner Activity Score	−0.06	0.776
Age at injury	Fracture healing time	0.33	0.103
Initial fracture displacement	Lysholm score	−0.36	0.073
Initial fracture displacement	Subjective IKDC score	−0.35	0.081
Initial fracture displacement	Tegner Activity Score	−0.45	0.022
Initial fracture displacement	Fracture healing time	0.35	0.084
Follow-up duration	Lysholm score	−0.01	0.978
Follow-up duration	Subjective IKDC score	−0.11	0.597
Follow-up duration	Tegner Activity Score	−0.03	0.878
Follow-up duration	Fracture healing time	0.10	0.626

Correlation coefficients (ρ) and corresponding *p* values describing the association between age at injury, initial fracture displacement, and follow-up duration with functional outcomes, including Lysholm score, subjective IKDC score, Tegner activity score, and fracture healing time.

Patients with residual anterior laxity demonstrated significantly lower Lysholm scores [median 98 [IQR, 95–99] vs. 100 [IQR, 98–100]; *p* = 0.023; rank-biserial correlation = −0.52] and lower Tegner Activity Scores [median 8 [IQR, 6–8] vs. 9 [IQR, 9–9]; *p* < 0.001; rank-biserial correlation = −0.78] than patients without residual laxity. No significant differences were observed in subjective IKDC score [median 97.3 (IQR 94.5–98.3) vs. 98.4 (IQR 96.8–99.4); *p* = 0.202; rank-biserial correlation = −0.30] or fracture healing time [median 40 days [IQR, 30–40] vs. 30 days [IQR, 30–40]; *p* = 0.164; rank-biserial correlation = 0.30]. The complete comparison between patients with and without residual anterior laxity is reported in Table 5.

**Table 5:** Comparison of clinical outcomes according to residual anterior knee laxity (Mann–Whitney U test).

Outcome	Lachman negative (*n* = 15) Median (IQR)	Lachman positive (*n* = 11) Median (IQR)	*p* value	Rank-biserial correlation
Lysholm score	100 (98–100)	98 (95–99)	0.023	−0.52
Subjective IKDC	98.4 (96.8–99.4)	97.3 (94.5–98.3)	0.202	−0.30
Tegner Activity Score	9 (9–9)	8 (6–8)	<0.001	−0.78
Healing Time (Days)	30 (30–40)	40 (30–40)	0.164	0.30

Median values with interquartile ranges (IQR) are reported for patients with and without residual anterior laxity (positive versus negative Lachman test). Between-group differences were analyzed using the Mann–Whitney U test. Effect sizes are expressed as rank-biserial correlation. Pre-injury and final Tegner activity scores were identical in every patient. Therefore, the between-group difference in final Tegner score reflects a difference in pre-injury activity level rather than a difference in postoperative recovery.

## Discussion

The principal finding of the present study is that the mini-open physeal-sparing technique provided reliable fracture healing, excellent functional outcomes, and high rates of return to sports at medium- to long-term follow-up. Fracture union was achieved in all patients, with no cases of growth disturbance, limb-length discrepancy, arthrofibrosis, or fixation failure. The majority of patients returned to their pre-injury level of activity.

The mean time to fracture union (35.8 ± 6.4 days) is consistent with previous clinical series reporting reliable and timely radiographic healing following surgical fixation of pediatric tibial eminence fractures (10, 23, 24). Restoration of knee motion was satisfactory, as no patient demonstrated persistent range-of-motion deficits at final follow-up. This is clinically relevant given that postoperative stiffness and arthrofibrosis remain among the most frequent complications after surgical treatment (9, 25).

Functional outcomes were excellent, with median subjective IKDC and Lysholm scores of 97.8 (IQR 96.5–99.4) and 99 (IQR 98–100), respectively. No significant difference was observed between pre-injury and final Tegner Activity Scores, indicating full recovery of pre-injury activity levels. These results are comparable to those reported in arthroscopic series, including D'Ambrosio et al. (26) Perugia et al. (27), and Tudisco et al. (28). Although direct comparison is not possible, the present findings suggest that mini-open fixation can achieve outcomes comparable to arthroscopic techniques.

Residual anterior laxity remains a common finding after tibial eminence fracture fixation (29). In our cohort, a positive Lachman test was observed in 42.3% of patients, although only one patient reported symptomatic instability. Patients with residual laxity showed significantly lower Lysholm scores than those without residual laxity, suggesting that objective knee laxity may be associated with subtle differences in patient-reported knee function. In contrast, subjective IKDC scores did not differ significantly between groups, indicating that residual laxity was not accompanied by a clinically meaningful reduction in patients' overall subjective perception of knee function. Although patients with residual laxity had lower Tegner Activity Scale scores at final follow-up, this finding reflects a lower pre-injury activity level rather than a poorer postoperative recovery, as all patients returned to their pre-injury Tegner level. Therefore, no association between residual laxity and the ability to resume the previous level of sports activity can be inferred from these data.

Similar observations have been reported in the literature. Kocher et al. (30) described persistent laxity despite excellent subjective outcomes after arthroscopic fixation. Wiley and Baxter (31) and Smith et al. (9) similarly reported a high prevalence of positive Lachman tests with limited or absent functional consequences.

The mechanisms underlying residual anterior laxity are likely multifactorial. Although tibial eminence fractures preserve ACL continuity, the initial trauma may cause microscopic fiber damage, plastic deformation, or elongation of the ligament. Higher-energy injury patterns, reflected by greater fracture displacement, may be associated with more severe ligamentous stress (23, 32). The absence of fixation failure or growth disturbance in our series supports the hypothesis that residual laxity is mainly related to the injury mechanism rather than surgical technique (33, 34).

Arthroscopic fixation is widely considered the reference technique for displaced tibial eminence fractures due to its ability to provide excellent visualization and address associated intra-articular lesions (22, 35–37). However, recent systematic reviews and meta-analyses have reported comparable clinical outcomes between arthroscopic and open techniques, with only slight advantages in objective stability in some series (10, 38). Overall, current evidence does not demonstrate clear superiority of one approach over the other, and treatment selection should be individualized (39, 40).

Within this context, the mini-open approach remains a valid alternative in selected cases. Direct visualization facilitates reduction, particularly in complex or comminuted fractures, and avoids the need for specialized arthroscopic instrumentation. Comparative studies have reported similar rates of fracture healing, complications, and functional outcomes between open and arthroscopic techniques (25, 39, 40). Therefore, surgical indication is more likely influenced by fracture pattern, surgeon experience, and institutional resources than by differences in intrinsic efficacy.

In addition, the mini-open technique offers practical advantages, including a shorter learning curve, reduced technical complexity, and potentially lower costs, which may facilitate its use in centers without dedicated pediatric arthroscopy expertise.

Nevertheless, the mini-open approach also has inherent disadvantages compared with arthroscopic fixation. It requires a larger skin incision, resulting in a more visible surgical scar and greater soft-tissue dissection. Consequently, postoperative pain and soft-tissue morbidity may be higher than with purely arthroscopic techniques. Furthermore, unlike arthroscopy, it does not allow comprehensive inspection and treatment of associated intra-articular lesions without extending the surgical exposure. Although these drawbacks should be acknowledged, current comparative studies have not demonstrated clinically meaningful differences in long-term functional outcomes between open and arthroscopic fixation.

Overall, the present study supports the mini-open physeal-sparing technique as a reliable option for displaced pediatric tibial eminence fractures. Rather than competing with arthroscopic approaches, it should be considered a complementary technique, particularly useful in specific fracture patterns or resource-limited settings.

## Strengths

The principal strengths of this study are the relatively long follow-up (mean 7.6 ± 2.9 years), the homogeneous surgical technique performed at a single institution, and the comprehensive clinical evaluation including objective and subjective functional outcomes.

## Limitations

This study has several limitations. First, its retrospective design and relatively small sample size limit the strength and generalizability of the findings. Second, the absence of a control group treated arthroscopically precludes direct comparison between surgical techniques. Third, objective instrumented measurements of anterior knee laxity, pivot-shift testing, and routine postoperative MRI assessment were not performed, potentially limiting the evaluation of residual instability and associated intra-articular pathology. In addition, standing long-leg radiographs and magnetic resonance imaging were obtained only when clinically indicated rather than routinely at final follow-up. Consequently, asymptomatic growth disturbances or subtle limb-length discrepancies may not have been detected in every patient, although no clinically relevant growth abnormalities were identified during follow-up. Finally, all procedures were performed at a single institution, which may limit external validity. Furthermore, the limited sample size reduced the statistical power of subgroup analyses and may have increased the risk of type II error. The absence of confidence intervals limits the precision with which the reported between-group differences can be interpreted. Moreover, in the absence of a control group, it is not possible to demonstrate either superiority or non-inferiority compared with the arthroscopic technique.

## Conclusion

The mini-open physeal-sparing technique appears to be a safe and effective treatment option for displaced pediatric tibial eminence fractures. In the present series, it provided reliable fracture healing, excellent functional outcomes, high rates of return to sport, and a low rate of complications at medium- to long-term follow-up. Although direct comparison with arthroscopic techniques is not possible in the absence of a control group, the results suggest that the mini-open approach remains a valuable alternative in selected cases, particularly when fracture characteristics or local resources limit the use of arthroscopic fixation.

## Data Availability

The raw data supporting the conclusions of this article will be made available by the authors, without undue reservation.
